# Is Cold Apparent Temperature Associated With the Hospitalizations for Osteoporotic Fractures in the Central Areas of Wuhan? A Time-Series Study

**DOI:** 10.3389/fpubh.2022.835286

**Published:** 2022-02-23

**Authors:** Faxue Zhang, Xupeng Zhang, Guangwen Zhou, Gaichan Zhao, Shijie Zhu, Xiaowei Zhang, Nan Xiang, Wei Zhu

**Affiliations:** ^1^Department of Occupational and Environmental Health, School of Public Health, Wuhan University, Wuhan, China; ^2^Department of Public Health, School of Public Health, Wuhan University, Wuhan, China; ^3^Department of Acupuncture and Orthopedics, Hubei University of Chinese Medicine, Wuhan, China

**Keywords:** apparent temperature, distributed lag non-linear model, osteoporotic fracture, time-series study, hospitalization

## Abstract

Osteoporosis is alarming problem due to aggravation of global aging, especially in China. Osteoporotic fracture (OF) is one of the most severe consequents of osteoporosis. Many previous studies found that environmental factors had adverse effects on human health. Cold temperature was associated with OF and bone metabolism in prior observational and experimental researches. However, few studies had been conducted on the acute effect of low temperature and OF. Data on daily meteorological factors and hospitalizations for OF were collected from Wuhan, China, between January 1, 2017 to December 24, 2019. Apparent temperature (AT), comprehensively considered a variety of environmental factors, was calculated by ambient temperature, relative humidity and wind speed. A generalized linear regression model combined with distributed lag non-linear regression model (DLNM) with quasi-Poisson link was used to explore the association between AT and the number of hospitalizations for OF. Subgroup analyses stratified by gender, age and the history of fracture were applied for detecting susceptible people. The exposure-response curve of AT and OF were generally U-shaped with lowest point at 25.8°C. The significant relationship of AT-OF existed only in cold effect (−2.0 vs. 25.8°C) while not in warm effect (37.0 vs. 25.8°C). Statistically significant risks of OF for cold effects were only found in females [RR = 1.12 (95%CI: 1.02, 1.24) at lag 2 day], aged <75 years old [RR = 1.18 (95%CI: 1.04, 1.33) and 1.17 (95%CI: 1.04, 1.33) at lag 2 and 3 days, respectively] and people with history of fracture [RR = 1.39 (95%CI: 1.02, 1.90) and 1.27 (95%CI: 1.05, 1.53) at lag 1 and 2 days, respectively]. The significant associations of AT on OF were only found in cold effect. The females, people aged <75 years and people with history of fracture possibly appeared to be more vulnerable. Public health departments should pay attention to the negative effect of cold AT and take measures in time.

## Introduction

Osteoporosis is a systemic skeletal disease characterized by a reduction in bone mass and micro-architectural deterioration of bone tissue (1). With the advancement of world population aging, the prevalence of osteoporosis was alarming and experts estimated that outnumbering 200 million people were suffering from osteoporosis (2, 3). Osteoporotic fractures (OF) is one of the most severe consequents of osteoporosis. According to the International Osteoporosis Foundation (IOF), there are nearly 9 million osteoporotic fractures worldwide every year, which means that a new case occurs every 3–4 s (4). One-third women and one-fifth men over the age of 50 years are predicted to suffer first time of osteoporotic fracture leading to limitations in quality of life (5, 6). All-cause death risks in the elderly with 1 years of bone fracture will increase 10–20%, and less than half of them were able to return to pre-fracture activity levels (7). About 300,000 people suffered from osteoporotic fracture accounting for nearly 2 million hospital bed days in the UK every year (8). In the United States of America, more than 2 million cases for osteoporotic fracture were reported and over 20 billion dollars of direct health cost were occurred in just 1 year (9). In Japan, family members as caregivers for patients with osteoporotic fracture leaded to over 20,000 dollars annual productivity loss per head (10).

Growing evidence pointed out that genetic, lifestyle, and environmental factors play roles in osteoporotic fracture to some extent (11–14). Fraenkel et al. found the daily rates of osteoporotic hip fractures in winter (1.1 cases per day) were significantly higher than summer (0.79 cases per day), autumn (0.90 cases per day) and spring (0.91 cases per day) (15). Compare with summer, the number of operations, length of hospital stays and cost for osteoporotic fractures of wrist in males were greatly increased in winter (8). A study conducted in Spain revealed the incidence of hip fracture was significantly associated with the coldest time of the year (16). Prior researchers also found that cold stimulus induced oxidative stress and inflammatory response (17, 18), which associated with exacerbating bone loss and enlarging the risks of osteoporotic fractures among the elderly (19, 20). An experimental study in New Zealand white rabbits found that short-term local cooling diminished bone healing by reducing bone blood flow (21). Compared with aging rats kept in a sedentary condition, the changes of bone mineral metabolism (such as the concentration of Ca, Mg, P, and key hormones) and bone mineral density were found in aging rats immersed in cold water (22). Exposures to low temperatures (−20~-15°C) in a short time affect bone growth by inducing premature arrest of the epiphyseal plate, destruction of the epiphysis, and reactive-endosteal and periosteal bone formation (23). The above literatures hint that cold temperature especially short-term exposure to low temperature might be a noteworthy factor affecting the occurrence of osteoporotic fractures. Myriad environmental epidemiological studies had chiefly chosen daily mean temperature and its variations as the indicators to investigate the relationship with health outcomes (24–26). However, apparent temperature (AT) comprehensively considered a variety of environmental factors (e.g., temperature, relative humidity, and wind speed) and can reflect the actual feeling of human body to the degree of heat, particularly in cold temperature environment (27, 28). Former studies found that AT was more sensitive to the association with mortality than other temperature variables (29, 30). Nevertheless, to the best of our knowledge, there was no study adapting AT as the indicator to investigate the health effects on osteoporotic fractures in China.

In this study, we aimed to use generalized linear model combined with distributed lag non-linear regression model to estimate the associations between short-term exposure to AT and the daily number of hospitalizations for osteoporotic fractures in urban population of Wuhan, China. Moreover, subgroup analyses were applied to detect potential susceptible population.

## Materials and Methods

### Study Area and Population

Wuhan, located in the middle reaches of Yangtze River, is the top 10 metropolises in China. The typical subtropical monsoon climate of Wuhan has extremely high temperature and much rain in the warm season and the opposite in the cold season. Wuhan Hospital of Traditional Chinese and Western Medicine is a large third-class public hospital situated in central of Wuhan (Supplementary Figure S1) with 4,260 beds and the number of outpatient/emergency visits and discharged patients are more than 3,000,000 and 100,000 per year, respectively (http://www.whyyy.com.cn/yygk/single/show/1.aspx).

### Data Collection

The data on hospitalization for osteoporotic fractures were retrieved from the health information system of Wuhan Hospital of Traditional Chinese and Western Medicine from January 1, 2017 to December 24, 2019. The medical information data after cleaning and quality controlling included the date of hospitalization, gender, age, the history of fracture and main diagnosis (code: M80) according to the International Classification of Diseases (10th revision). We summarized the number of hospitalizations for osteoporotic fractures in each calendar day to establish a new database for analyzing.

Data on meteorological factors in this study period were collected from China National Meteorological Science Data Center (http://data.cma.cn/), including daily mean temperature, relative humidity, wind velocity, sunshine duration and rainfall. Daily mean fine particulate matter (PM_2.5_), inhalable particles (PM_10_), sulfur dioxide (SO_2_), nitrogen dioxide (NO_2_), carbon monoxide(CO), and ozone (O_3_) were collected at the nearest monitoring station from Wuhan Environmental Protection Bureau (http://hbj.wuhan.gov.cn/). Apparent temperature (AT) was calculated by some meteorological factors as follows (28).


(1) $$\begin{array}{r} wvp=\frac{Rh}{100}\times 6.105\times e^{\frac{17.27\times Ta}{237.7+Ta}} \end{array}$$

(2) $$\begin{array}{r} AT=Ta+0.33\times wvp-0.70\times WS-4.00 \end{array}$$

Where *wvp* was water vapor pressure (hPa); *Rh* was relative humidity (%); *Ta* was ambient temperature (°C); *WS* was wind speed (m/s).

### Statistical Analysis

Demographic and meteorological factors were described as mean, standard deviation, and quantile. The Spearman analyses were carried out to estimate the correlations of meteorological factors. In this study, a generalized linear regression model combined with distributed lag non-linear regression model (DLNM) with quasi-Poisson link was used to explore the associations between AT and the number of hospitalizations for osteoporotic fractures. Natural cubic smoothing splines with degrees of freedom (dfs) of 5 and 3 were used for exposure-response and lag-response associations in cross-basis function, respectively (31). The effect of apparent temperature exposure was speculated using a natural cubic spline with four internal equally spaced values knots (20th, 40th, 60th, and 80th) and the lagged-response association was modeled with a natural cubic spline with 1 internal knot at equally spaced value (31). Due to the potential delayed effects of AT on the number of hospitalizations for osteoporotic fractures, after exploratory test (Supplementary Figures S2, S3), this study set up 10 days as maximum lag (31). Covariates included time, rainfall, sunshine duration, public holiday (PH), and day of week (DOW) were incorporate in the final model as follows (32–38):


$$\begin{array}{l} \text{Log}\left(\text{Yt}\right)\;=\text{ α}+\text{β}\times {\text{AT}}_{\text{t},\text{l}}+ns\left(time,\;df\right)+ns\left(rainfall,\;df\right)\\ \;+ns\left(sunshine\;duration,\;df\right)+\gamma \times \text{PH}+\delta \times \text{DOW} \end{array}$$

Where Yt is the daily number of hospitalizations for osteoporotic fractures in day t; α is the intercept of the model; *AT*_*t, l*_ is the matrix of the daily mean apparent temperature obtained from the cross-basis function in the DLNM; β is the coefficient vector of matrix of *AT*_*t, l*_, and l was the lag days; *ns* is natural cubic smoothing function for the nonlinear variables such as time, rainfall, and sunshine duration; *df* means the degree of freedom; According to previous literature and pre-analysis results (Supplementary Figures S4, S5), we defined 1 df per year for time trends and 3 df per year for daily mean rainfall and sunshine duration in the final model, respectively (31, 37).

According to preliminary analyses, optimum apparent temperature (OAT) was 25.8°C, which defined as the lowest risks of AT-OF in the exposure-response curve (Supplementary Figure S6). Cold and warm effects were defined as the effect values of 2.5th (−2°C) and 97.5th (37°C) AT percentiles compared with OAT, respectively, to exhibit the associations between AT and osteoporotic fractures. We furtherly analyzed the association between AT and the number of hospitalizations for osteoporotic fractures in different gender (males and females) age (0–75 and over 75 years old) and the history of fracture (yes and no) groups. The potential interaction in subgroups (gender, age, and the history of fracture) were analyzed by *Z*-test (39). After estimating the health effects of AT in the initial model, four sensitivity analyses were conducted to assess the robustness of effects of AT on the number of hospitalizations for osteoporotic fractures. The first was compare the result of the cold effect of absolute temperature [optimum absolute temperature was 297.1 K (for the convenience of comparison, the temperature scale of Celsius degree is adopted, so 297.1 K = 24.0°C) (Supplementary Figure S7)] and AT; The second was adding six air pollutants (PM_2.5_, PM_10_, SO_2_, NO_2_, CO, and O_3_) in the model one by one; The third was changing the maximum lag days (from 10 to 9 or 11) and dfs (for AT from 5 to 4 or 6, for lag from 3 to 2 or 4) for the cross-basis function of daily mean temperature; The last one was changing the dfs for time (from 1 to 2 or 3), rainfall and sunshine duration (from 3 to 2 or 4) to examine the robustness of the results in our study.

Consistent with previous studies, the effects of AT were reported as relative risk (RR) and 95% confidence interval (CI). All statistical analyses were conducted by “dlnm” and “splines” packages in R software (version 4.0.5). Results with a 2-sided and *p* < 0.05 were statistically significant.

## Results

Table 1 shows the descriptive statistics of hospitalization data due to osteoporotic fracture (OF) as well as daily average meteorological factors in Wuhan, from January 1, 2017 to December 24, 2019. A total of 1,488 hospitalizations for osteoporotic fracture were collected from Wuhan Hospital of Traditional Chinese and Western Medicine. Males and patients with a history of fracture accounted for 22.04 and 20.56% of the whole patients. Approximately half of the patients were aged 75+ years. For meteorological factors, the daily mean AT and temperature were 18.5 and 17.5°C, respectively. During the study period, the daily mean relative humidity, wind speed, sunshine duration, and rainfall were 79.0%, 1.6 m/s, 4.6 h, and 3.0 mm, separately.

**Table 1:** The descriptions of hospitalizations for osteoporotic fracture and meteorological factors.

	**All (%)**	**Mean**	**SD**	**Min**	**P25**	**Median**	**P75**	**Max**
**Hospitalization visits**	1,488	1.4	1.3	0.0	0.0	1.0	2.0	8.0
**Gender**								
Male	328 (22.04)	0.3	0.6	0.0	0.0	0.0	0.0	3.0
Female	1,158 (77.82)	1.1	1.1	0.0	0.0	1.0	2.0	7.0
**Age (years)**								
<75	755 (50.74)	0.7	0.9	0.0	0.0	0.0	1.0	6.0
75+	733 (49.26)	0.7	0.9	0.0	0.0	0.0	1.0	5.0
**History of fracture**								
Yes	306 (20.56)	0.3	0.6	0.0	0.0	0.0	0.0	5.0
No	1,180 (79.30)	1.1	1.1	0.0	0.0	1.0	2.0	8.0
**Meteorological factors**								
AT (°C)	–	18.5	11.9	−7.1	7.9	18.9	28.7	40.3
Temperature (°C)	–	17.5	9.4	−3.8	9.2	18.1	25.8	33.9
Relative humidity (%)	–	79.0	10.6	41.0	72.0	79.0	87.0	100.0
Wind speed (m/s)	–	1.6	0.9	0.2	0.9	1.4	2.1	6.3
Sunshine duration (h)	–	4.6	4.2	0.0	0.0	4.4	8.4	13.0
Rainfall (mm)	–	3.0	9.4	0.0	0.0	0.0	1.1	174.7

*Min, minimum; Max, maximum; SD, standard deviation*.

Supplementary Figure S8 shows the variations of daily mean apparent temperature, temperature, relative humidity, and wind speed from January 1, 2017 to December 24, 2019. Similar trends were observed between AT and temperature which were both reaching the peak in summer and dropping into low ebb in winter. Relatively higher values of AT than temperature were mainly found in warm season. However, no obvious trend changes were observed in relative humidity and wind speed. Spearman correlation coefficients between meteorological variables were shown in Supplementary Figure S9. The relationships of AT and relative humidity, wind speed, and rainfall were almost negative. The largest positive correlation with statistical significance were found between AT and temperature (correlation coefficient = 0.998, *P* < 0.01), as seen in Supplementary Table S1.

**Figure 1:**
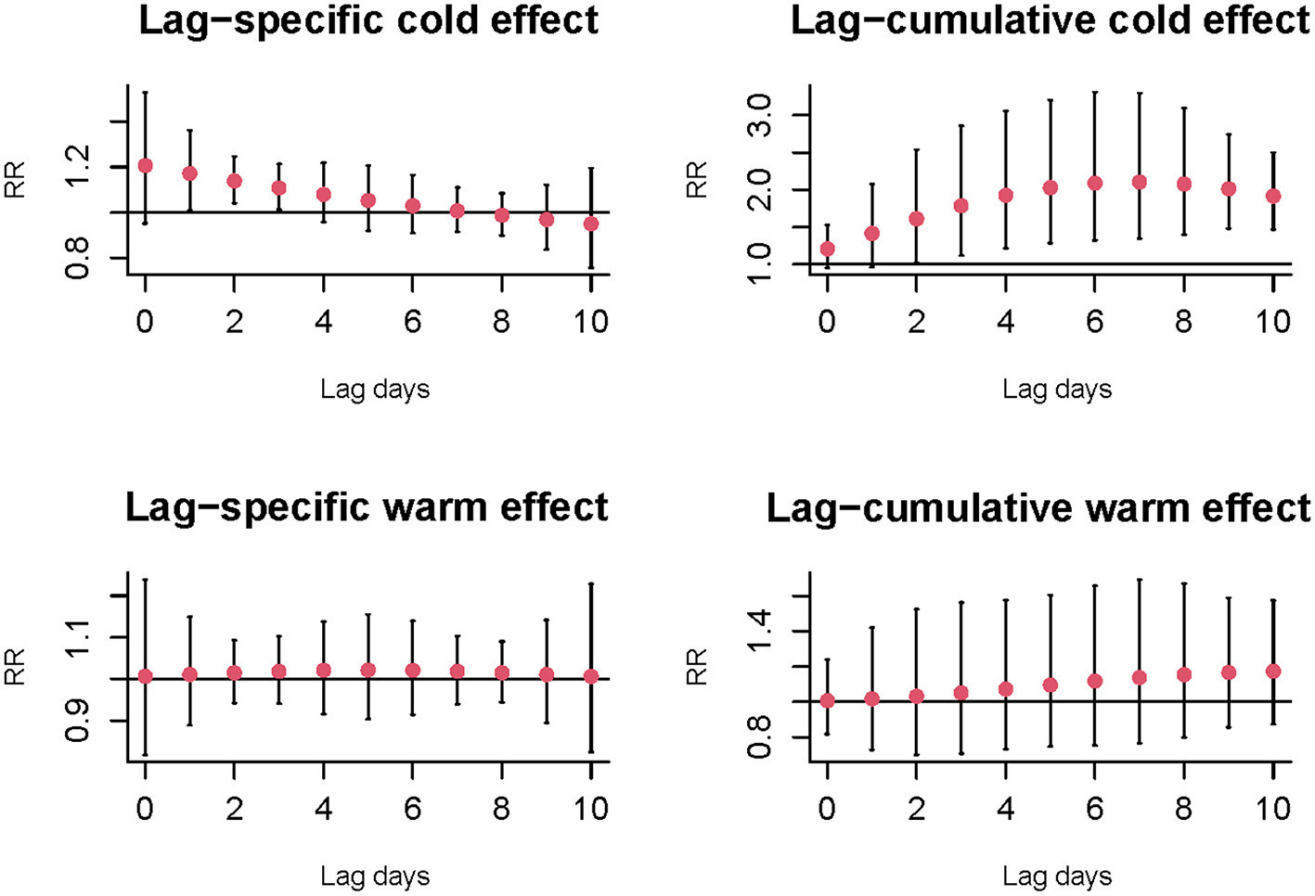
The relative risks and 95%CI of cold (−2.0 vs. 25.8°C) and warm effect (37.0 vs. 25.8°C) on the number of hospitalizations for osteoporotic fractures at single and cumulative lag days.

Supplementary Figure S6 illustrates the exposure-response curve between apparent temperature and the relative risks of hospitalization visits for osteoporotic fractures at lag 10 day. In general, a wide U-shape with large opening and lowest point at 25.8°C was observed, which corresponding to the minimal risks of AT. Figure 1 shows the estimated values with 95%CI of cold and warm effects on the number of hospitalizations for osteoporotic fractures. Statistically significant relationships of lag-response were only found in cold effects. The largest RR values (−2.0 vs. 25.8°C) were 1.17 (95%CI: 1.01, 1.36) at lag 1 day in single-lag effects and 2.10 (95%CI: 1.34, 3.30) along 0–7 days in cumulative-lag effects (Supplementary Table S2). No associations between warm effect (37.0 vs. 25.8°C) and osteoporotic fractures were identified in single and cumulative lag days.

**Figure 2:**
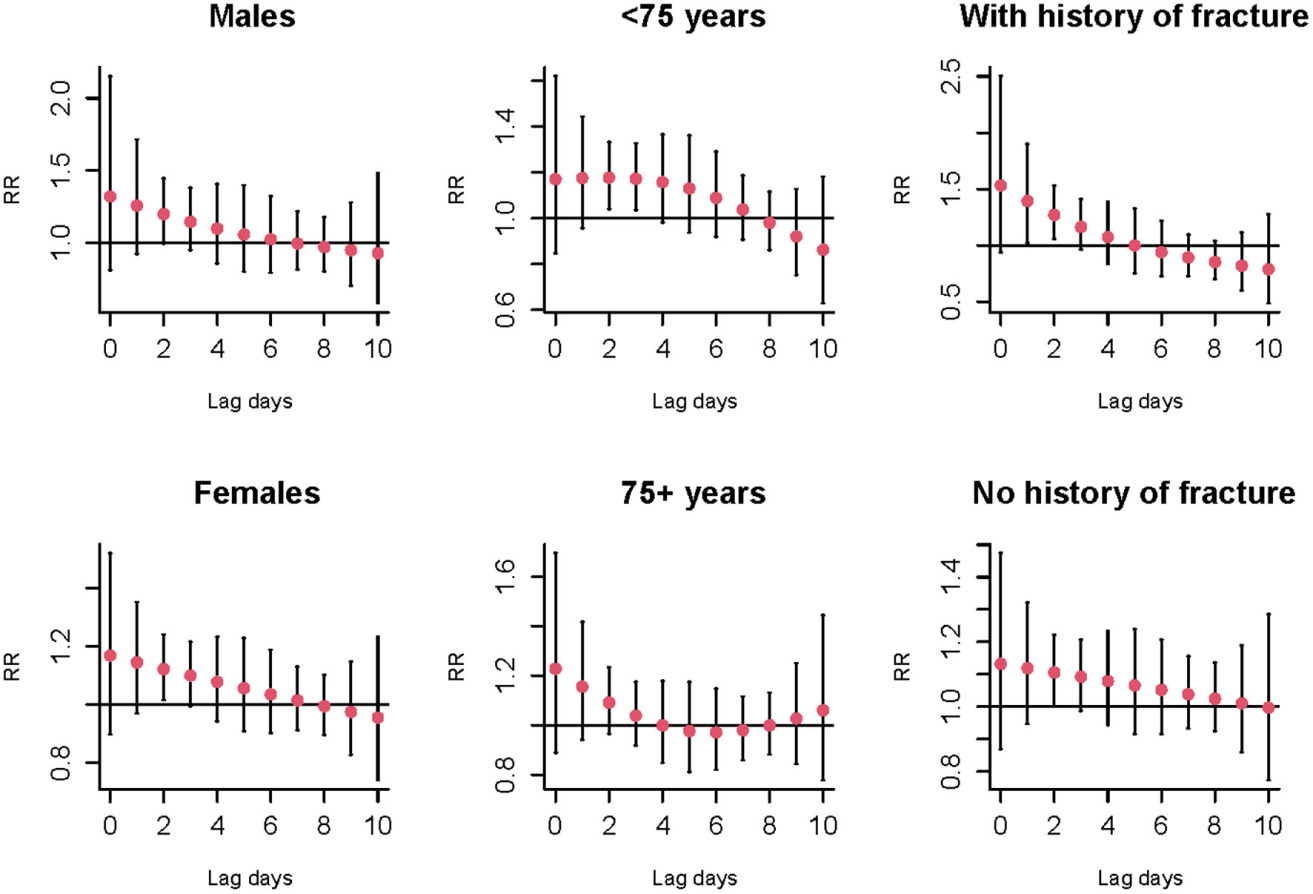
The relative risks and 95%CI of cold effect (−2.0 vs. 25.8°C) on the number of hospitalizations for osteoporotic fractures stratified by gender, age, and history of fracture at different lag days.

**Figure 3:**
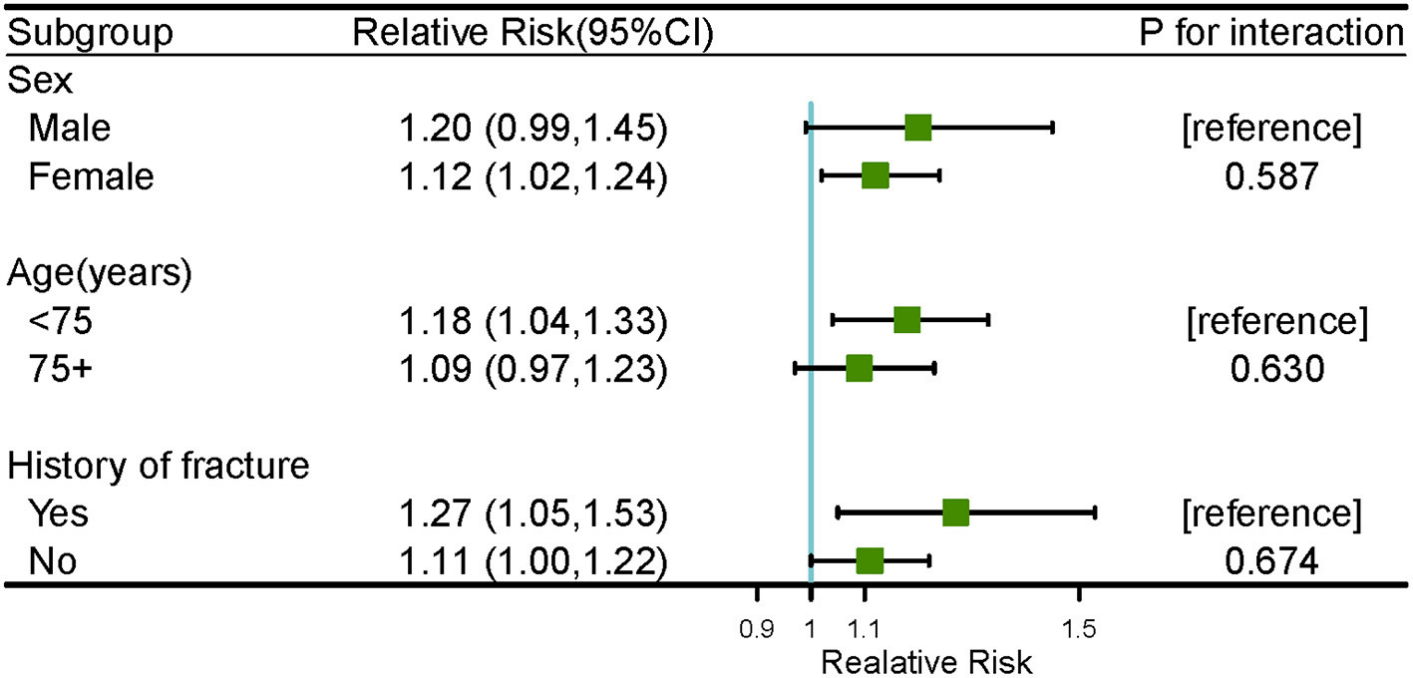
The results of *Z*-test for cold effect (−2.0 vs. 25.8°C) on the number of hospitalizations for osteoporotic fractures within subgroup analyses at lag 2 day.

Figure 2 shows the cold effects on the hospitalizations for osteoporotic fractures stratified by gender, age, and history of fracture. Significant risks of osteoporotic fractures for cold effects were only found in females [RR = 1.12 (95%CI: 1.02, 1.24) at lag 2 day], aged <75 years old [RR = 1.18 (95%CI: 1.04, 1.33) and 1.17 (95%CI: 1.04, 1.33) at lag 2 and 3 days, respectively] and history of fracture [RR = 1.39 (95%CI: 1.02, 1.90) and 1.27 (95%CI: 1.05, 1.53) at lag 1 and 2 days, respectively] (Supplementary Table S3). No significant associations of warm effects and osteoporotic fractures were observed in subgroup analyses, as seen in Supplementary Figure S10 and Supplementary Table S4. The results of *Z*-test for the differences within each subgroup for the risks of cold effects at lag 2 day were shown in Figure 3. No statistically significant differences were found in gender, age, and history of fracture subgroups (*P* for interaction were 0.587, 0.630, and 0.674, respectively).

Table 2 shows the relative risks and 95%CI of cold effect of absolute temperature and apparent temperature on the number of hospitalizations for osteoporotic fractures at lag 2 day. Similar effect patterns were observed in this two indicators. However, compared with absolute temperature, AT might be more sensitive to people who had the history of fracture [RR = 1.27, 95% CI: (1.05, 1.53)]. As seen in Table 3, though the values of RR were slightly changed after adjusting for air pollutants one by one, the cold effect of AT remained statistically significant. Supplementary Table S5 summaries the relative risks and 95%CI of osteoporotic fractures for cold effects at lag 2 day in sensitive analyses. After changing the parameters for AT and maximum lag days in cross-basis functions and the degrees of free for time (from 1 to 2 or 3), rainfall and sunshine duration (from 2 to 3 or 4) in the initial model, most of RR values also remained statistically significant, which meant our results and model were robust.

**Table 2:** The relative risks and 95%CI of cold effect of absolute temperature and apparent temperature on the number of hospitalizations for osteoporotic fractures at lag 2 day.

	Absolute temperature*	Apparent temperature**
	**Cold effect**	**Cold effect**
**All**	**1.14 (1.04, 1.25)**	**1.14 (1.04, 1.25)**
**Gender**		
Male	1.19 (0.97, 1.45)	1.20 (0.99, 1.45)
Female	**1.12 (1.01, 1.24)**	**1.12 (1.02, 1.24)**
**Age**		
<75	**1.19 (1.06, 1.35)**	**1.18 (1.04, 1.33)**
75+	1.12 (0.98, 1.29)	1.09 (0.97, 1.23)
**History of fracture**		
Yes	1.22 (0.98, 1.50)	**1.27 (1.05, 1.53)**
No	1.10 (1.00, 1.22)	1.11 (1.00, 1.22)

Bold represents statistically significant; *Cold effect (0.5 vs. 24.0°C) of absolute temperature; **Cold effect (−2.0 vs. 25.8°C) of apparent temperature.

**Table 3:** The relative risks (95% CI) of cold effect after adjusting for air pollutants at lag 2 day.

	**RR (95% CI)**
**Final model**	**1.139 (1.041, 1.247)**
+PM_2.5_	**1.135 (1.034, 1.246)**
+PM_10_	**1.129 (1.028, 1.241)**
+SO_2_	**1.138 (1.040, 1.246)**
+NO_2_	**1.137 (1.040, 1.243)**
+CO	**1.136 (1.035, 1.246)**
+O_3_	**1.115 (1.014, 1.226)**

*Bold represents statistically significant*.

## Discussion

In this study, we applied generalized linear model combined with DLNM to explore the associations of AT and osteoporotic fractures in urban population of Wuhan, China. To our knowledge, this study was the first to analyze the effect of AT on hospitalizations for osteoporotic fractures in China. For all groups, only cold effect of AT had significantly nonlinear and delayed effects on hospital admissions for osteoporotic fractures was found. In addition, the female patients, patients aged <75 years and patients with history of fracture appeared to be more vulnerable to cold effect of AT since significant correlations were only observed in their subgroups, respectively. These findings might provide evidence that more targeted and effective preventive measures conducted by relative departments need to be adopt to these susceptible people.

We found significant association between low AT and the hospitalizations for osteoporotic fractures in the current study. In view of the highly identity between AT and air temperature, the cold effect was consistent with previous studies focused on temperature and fractures (8, 15, 40). As early as 2004, a Hungarian study pointed out that the prevalence of hypovitaminosis D during spring (71%) was higher than that in summer (46.3%) (35), which may affect bone health due to the limited absorption of calcium increased bone resorption (41). Lower AT usually accompanied by ice and snow weather may also lead to falls in the elderly, increasing the incidence of osteoporotic fractures (16, 42). In Australia, lower daily temperatures were significantly associated with higher hospitalization rates for fall related hip fractures in people aged over 75 years old (40). However, the significant difference of incidence of fractures in different temperatures, temperature changes, seasons and months were not observed in Southern England (33). The possible explanation might be that the climate and people's tolerance for temperature were different in various regions.

The specific biological mechanisms of the cold effect of AT and the hospitalizations for osteoporotic fractures were not clear yet. According to available studies, some possible mechanisms were exhibited in the followings: First, in cold environment, the duration of sunshine is usually short and the available vitamin D is limited, which aggravates the occurrence of osteoporosis and leads to osteoporotic fractures (35). Second, physical activity was proved to be linked with high bone mineral density, superior neuromuscular function, while lower temperature was a cause of reduced physical activity (36, 43). Third, lower temperature leads to impaired flexibility, decreased neuromuscular function and shortened response time, or has an impact on hemodynamic status and blood pressure, increasing the risk of falls (34, 44, 45). Fourth, lower temperature was associated with higher fracture rates only in people without influenza vaccination suggesting that influenza outbreaks may increase the risk of hip fractures (15). Finally, mice experiments revealed that cold stress could reduce the proliferation of bone marrow mesenchymal stem cells (BMMSCs), which were the main source of osteoblasts (46, 47).

Our results showed that the females seemed to be more vulnerable to cold effect than males. The subgroup analysis of gender was similar with some earlier studies focusing on the relationship between temperature and osteoporotic fractures (8, 35). A former study found the prevalence of low vitamin D disease associated with high incidence of osteoporosis was higher in females than males (35). Estrogen, promoted the proliferation and differentiation of osteoblasts by binding with estrogen receptor (ER) on osteoblasts, played an important role in bone metabolism (48). The decrease of estrogen secretion caused by menopause in women with higher plasma dipeptide peptide 4 (DPP4) levels will lead to an increase in bone turnover and the prevalence of osteoporotic fractures (49). However, the significant association between low temperature and fracture was also observed in males, which was thought to represent a vulnerable fracture in males who spend more time outdoors than women (8). More studies were warranted to provide evidence about whether females with postmenopausal osteoporosis have lower tolerance of cold effect.

In this study, the connection between cold effect of AT and risk of hospitalizations for osteoporotic fractures was only found in people aged <75 years. Levy et al. (32) reported similar findings with our study that the effect of cold weather on hip fracture rates was highest in younger persons. Younger people might have more exposure opportunities for cold effect of AT due to longer duration of time in outdoors, so that they would have a higher risk of hospitalization for osteoporotic fractures. However, significant association between lower daily air temperature with higher fall-related hip fracture hospitalizations in 75+ years old was also found in an Australian study (40). The inconsistent outcomes in these studies may be caused by different populations and lifestyle.

In single-day lag analysis of this study, the risk of hospitalizations for osteoporotic fractures associated with low AT were only found in people with a history of fractures. Former studies pointed out that 23% of subsequent fractures occur within 1 year and 54% within 5 years after the first fracture (50, 51). A meta-analysis found that regardless of the type of initial fracture, the risk of re-fracture was about twice that of people without a history of fracture, especially higher in patients with postmenopausal osteoporotic fractures (52). It is plausible that with bone mass rapidly lost due to the bed-rest and immobilization during the acute period after the fracture, the bone mineral density will continue to decline in following 3–6 months (53). This may explain the people with a history of fracture were more vulnerable to cold effect and increasing the risk of hospitalizations for osteoporotic fractures.

Prior epidemiological studies pointed out that warm effect of AT was associated with all-cause mortality and hospital admissions for cardiovascular diseases (54–56). However, no statistical significance was found in the warm effect of AT on the risk of hospitalizations for osteoporotic fractures in this study. A study conducted in Taiwan observed significant negative associations between ambient temperature and hip fractures, which indicated that higher temperature may have a protective effect in fractures and will not increase the risk of hospitalizations for osteoporotic fractures (36). The difference findings may be due to people living in different climatic environments with different tolerances to temperature. In addition, there may be certain time temporal trends in the association of ambient temperature and health (57, 58).

There were some strengths in this study. First, AT, the new comprehensive index about heat, may be more realistic and objective as an early warning indicator than temperature itself, which is widely accepted in earlier studies (37, 59, 60). Second, we compared the differences of subgroups affected by the cold effect. It may be conducive to more focused policy implementation in relevant prevention work. And the difference between subgroups with or without fracture history was also mentioned in similar studies for the first time. Third, this paper found a significant relationship between AT and osteoporotic fractures. This may provide a new idea for the following related research. Finally, the application of DLNM was scientific dealing with non-linear exposure–response relationships and delayed effects (61, 62).

Several limitations in this study should be recognized. First, meteorological data was obtained from one fixed-site meteorological monitoring station and the use of mean AT as proxies for personal exposure were inevitably expected to cause exposure misclassification (63, 64). More accurate ways, for example, estimating individual's exposure by simulating and calculating using satellite data based on home address, were warranted. Second, we could not acquire the data of individual diet, medication and physical activity, which might be confounding factors of the association of AT and bone strength. Lastly, the region we selected was limited to Wuhan, and the sample of hospitalizations is relatively small, lacking generalizability to other populations.

Findings of this study may provide some references for public health policy. In this study, AT has a cold effect on the risk of hospitalizations for osteoporotic fractures. Early warning according to the predicted meteorological data can be formulate by public health department. For example, the female patients, patients aged <75 years and patients with history of fracture can be checked or inquired regularly through the community or hospital system. Especially in China, where the phenomenon of empty nesters is very common, the mortality rate can reach 21% within 1 year once osteoporotic fractures occur (65). It is necessary to identify potential patients and take precautions earlier according to the correlation between AT and osteoporotic fractures. And early medical allocation is also theoretically feasible. The effect of other possible risk factors needs to be addressed by future studies. It is hoped that this study can draw more researchers' attention to make more improvement and exploration.

## Conclusions

In conclusion, we investigate the effects of AT on the daily hospitalizations for osteoporotic fractures from 2017 to 2019 in urban population of Wuhan, China. Results in this study showed that short-term exposure to cold effect of AT was linked with the increased risk of hospitalizations for osteoporotic fractures at Wuhan Hospital of Traditional Chinese and Western Medicine, which meant AT was a novel indicator to estimate the relationship between environment and disease. Female patients, patients aged <75 years and patients with history of fracture possibly appeared to be more vulnerable to cold effect of AT. Consequently, public health departments should consider not only the health impact of daily mean temperature, but also AT, to formulate better preventive measures.

## Data Availability Statement

The original contributions presented in the study are included in the article/Supplementary Material, further inquiries can be directed to the corresponding author/s.

## Ethics Statement

This study was approved by the Ethics Committee of Wuhan University.

## Author Contributions

WZ and NX: conceived and designed the study. GZho, GZha, and SZ: collected and cleaned the data. FZ and XuZ: performed the data analysis and drafted the manuscript. WZ and XiZ: helped to revise the manuscript. All authors read and approved the final manuscript.

## Funding

This study was funded by grants from the National Natural Science Foundation of China (No. 82074416).

## Conflict of Interest

The authors declare that the research was conducted in the absence of any commercial or financial relationships that could be construed as a potential conflict of interest.

## Publisher's Note

All claims expressed in this article are solely those of the authors and do not necessarily represent those of their affiliated organizations, or those of the publisher, the editors and the reviewers. Any product that may be evaluated in this article, or claim that may be made by its manufacturer, is not guaranteed or endorsed by the publisher.
