# Supplementary material for: Is Cold Apparent Temperature Associated With the Hospitalizations for Osteoporotic Fractures in the Central Areas of Wuhan? A Time-Series Study
Source: Front Public Health. 2022 Feb 23;10:835286. doi: 10.3389/fpubh.2022.835286 (PMC8904880; doi:10.3389/fpubh.2022.835286)
Supplement: Supplementary file 1 [file Data_Sheet_1.docx]

Supplementary Material

Is cold apparent temperature associated with the hospitalizations for osteoporotic fractures in the central areas of Wuhan? A time-series study

**Faxue Zhang ^1^**^†^**, Xupeng Zhang ^2^**^†^**, Guangwen Zhou ^3^, Gaichan Zhao ^2^, Shijie Zhu ^1^, Xiaowei Zhang ^1^, Nan Xiang ^3 *^, Wei Zhu ^1 *^**

^1^ Department of Occupational and Environmental Health, School of Public Health, Wuhan University, Wuhan 430071, China

^2^ Department of Public Health, School of Public Health, Wuhan University, Wuhan 430071, China

^3^ Department of Acupuncture and Orthopedics, Hubei University of Chinese Medicine, Wuhan 430065, China

*** Correspondence:**Nan Xiang

xiangnanhucm@163.com

Wei Zhu

weizhu@whu.edu.cn

^†^These authors have contributed equally to this work and share first authorship.

Keywords: apparent temperature, distributed lag non-linear model, osteoporotic fracture, time-series study, hospitalization.

Table of contents

**Table S1.** The Spearman correlation of meteorological variable.

**Table S2.** The relative risks and 95%CI of cold (-2.0 ℃ vs 25.8 ℃) and warm effects (37.0 ℃ vs 25.8 ℃) on the number of hospitalizations for osteoporotic fractures at single and cumulative lag days.

**Table S3.** The relative risks and 95%CI of cold effect (-2.0 ℃ vs 25.8 ℃) on the number of hospitalizations for osteoporotic fractures stratified by gender, age and history of fracture at single-lag days.

**Table S4.** The relative risks and 95%CI of warm effect (37.0 ℃ vs 25.8 ℃) on the number of hospitalizations for osteoporotic fractures stratified by gender, age and history of fracture at single-lag days.

**Table S5.** The relative risk with 95%CI in hospitalizations for osteoporotic fractures of cold effect (-2.0℃ vs 25.8℃) at lag 2 day by changing different lag or degree of freedom (df) values for parameters of cross-basis function, calendar time, rainfall, and sunshine duration.

**Figure S1.**  The location and main service scope of Wuhan Hospital of Traditional Chinese and Western Medicine.

**Figure S2.** The AIC at different lag day.

**Figure S3.** Cumulative association and AT distribution of OF at different lag days.

**Figure S4.** The AIC at different df of time.

**Figure S5.** Cumulative association and AT distribution of OF at different df for time trend.

**Figure S6.** The exposure-response curve of apparent temperature and the hospitalizations for osteoporotic fractures at lag 0-10 days

**Figure S7.** The exposure-response curve of absolute temperature and the hospitalizations for osteoporotic fractures at lag 0-10 days.

**Figure S8.** The variations of daily meteorological factors from 2017 to 2019 in Wuhan, China.

**Figure S9.** The spearman correlation coefficients between meteorological factors.

**Figure S10.** The relative risks and 95%CI of warm effect (37℃ vs 25.8℃) on the number of hospitalizations for osteoporotic fractures stratified by gender, age and history of fracture at different lag days.

**Table S1.** The Spearman correlation of meteorological variables.

|  | AT | Temp | RH | WS | SD | RF |
| --- | --- | --- | --- | --- | --- | --- |
| AT | 1.000 | 0.998** | -0.128** | -0.002 | 0.431** | -0.074* |
| Temp |  | 1.000 | -0.164** | 0.047 | 0.435** | -0.080** |
| RH |  |  | 1.000 | -0.048 | -0.524** | 0.578** |
| WS |  |  |  | 1.000 | -0.221** | 0.296** |
| SD | |  |  |  | 1.000 | -0.521** |
| RF |  |  |  |  |  | 1.000 |

**P value<0.01, *P value<0.05, *AT: apparent temperature, Temp: temperature, RH: relative humidity, WS: wind speed, SD: sunshine duration, RF: rainfall*

**Table S2.** The relative risks and 95%CI of cold (-2.0℃ vs 25.8℃) and warm effect (37.0℃ vs 25.8℃) on the number of hospitalizations for osteoporotic fractures at single and cumulative lag days.

| Lag day | Relative Risk(95%CI) | |
| --- | --- | --- |
|  | Cold effect | Warm effect |
| Lag 0 | 1.21 (0.95,1.53) | 1.01 (0.82,1.24) |
| Lag 1 | **1.17 (1.01,1.36)** | 1.01 (0.89,1.15) |
| Lag 2 | **1.14 (1.04,1.25)** | 1.02 (0.94,1.09) |
| Lag 3 | **1.11 (1.01,1.21)** | 1.02 (0.94,1.10) |
| Lag 4 | 1.08 (0.96,1.22) | 1.02 (0.92,1.14) |
| Lag 5 | 1.05 (0.92,1.21) | 1.02 (0.90,1.16) |
| Lag 6 | 1.03 (0.91,1.17) | 1.02 (0.91,1.14) |
| Lag 7 | 1.01 (0.92,1.11) | 1.02 (0.94,1.10) |
| Lag 8 | 0.99 (0.90,1.08) | 1.02 (0.95,1.09) |
| Lag 9 | 0.97 (0.84,1.12) | 1.01 (0.89,1.14) |
| Lag 10 | 0.95 (0.76,1.20) | 1.01 (0.82,1.23) |
| Lag 0-1 | 1.41 (0.96,2.08) | 1.02 (0.73,1.42) |
| Lag 0-2 | **1.61 (1.02,2.54)** | 1.03 (0.70,1.52) |
| Lag 0-3 | **1.79 (1.12,2.86)** | 1.05 (0.71,1.56) |
| Lag 0-4 | **1.93 (1.21,3.06)** | 1.07 (0.73,1.58) |
| Lag 0-5 | **2.03 (1.29,3.20)** | 1.10 (0.75,1.61) |
| Lag 0-6 | **2.09 (1.32,3.31)** | 1.12 (0.75,1.66) |
| Lag 0-7 | **2.10 (1.34,3.30)** | 1.14 (0.76,1.69) |
| Lag 0-8 | **2.08 (1.39,3.10)** | 1.15 (0.80,1.67) |
| Lag 0-9 | **2.01 (1.48,2.74)** | 1.17 (0.86,1.59) |
| Lag 0-10 | **1.91 (1.47,2.50)** | 1.17 (0.87,1.58) |

Note: Bold results are statistically significant (p<0.05)

**Table S3.** The relative risks and 95%CI of cold effect (-2.0℃ vs 25.8℃) on the number of hospitalizations for osteoporotic fractures stratified by gender, age and history of fracture at single-lag days.

| Lag day | Gender | |  | Age(years) | |  | History of fracture | |
| --- | --- | --- | --- | --- | --- | --- | --- | --- |
|  | Male | Female |  | <75 | ≥75 |  | Yes | No |
| lag0 | 1.32 (0.81,2.15) | 1.17 (0.90,1.52) | | 1.17 (0.85,1.62) | 1.23 (0.89,1.70) | | 1.53 (0.94,2.50) | 1.13 (0.87,1.47) |
| lag1 | 1.26 (0.92,1.72) | 1.15 (0.97,1.35) | | 1.18 (0.96,1.44) | 1.16 (0.94,1.42) | | **1.39 (1.02,1.90)** | 1.12 (0.95,1.32) |
| lag2 | 1.20 (0.99,1.45) | **1.12 (1.02,1.24)** | | **1.18 (1.04,1.33)** | 1.09 (0.97,1.23) | | **1.27 (1.05,1.53)** | 1.11 (1.00,1.22) |
| lag3 | 1.15 (0.95,1.38) | 1.10 (0.99,1.22) | | **1.17 (1.04,1.33)** | 1.04 (0.92,1.18) | | 1.16 (0.96,1.41) | 1.09 (0.99,1.21) |
| lag4 | 1.10 (0.86,1.41) | 1.08 (0.94,1.23) | | 1.16 (0.98,1.37) | 1.00 (0.85,1.18) | | 1.07 (0.83,1.38) | 1.08 (0.94,1.23) |
| lag5 | 1.06 (0.80,1.40) | 1.06 (0.91,1.23) | | 1.13 (0.94,1.36) | 0.98 (0.81,1.18) | | 1.00 (0.75,1.33) | 1.07 (0.92,1.24) |
| lag6 | 1.02 (0.79,1.32) | 1.04 (0.90,1.19) | | 1.09 (0.92,1.29) | 0.97 (0.82,1.15) | | 0.94 (0.72,1.22) | 1.05 (0.92,1.21) |
| lag7 | 0.99 (0.81,1.22) | 1.01 (0.91,1.13) | | 1.04 (0.91,1.19) | 0.98 (0.86,1.12) | | 0.89 (0.73,1.10) | 1.04 (0.93,1.16) |
| lag8 | 0.97 (0.80,1.18) | 0.99 (0.90,1.10) | | 0.98 (0.86,1.12) | 1.00 (0.88,1.13) | | 0.85 (0.70,1.04) | 1.03 (0.92,1.14) |
| lag9 | 0.95 (0.70,1.28) | 0.97 (0.83,1.15) | | 0.92 (0.75,1.13) | 1.03 (0.84,1.25) | | 0.82 (0.60,1.12) | 1.01 (0.86,1.19) |
| lag10 | 0.93 (0.58,1.48) | 0.96 (0.74,1.23) | | 0.86 (0.63,1.18) | 1.06 (0.78,1.44) | | 0.79 (0.49,1.28) | 1.00 (0.77,1.29) |

Note: Bold results are statistically significant (p<0.05)

**Table S4.** The relative risks and 95%CI of warm effect (37.0℃ vs 25.8℃) on the number of hospitalizations for osteoporotic fractures stratified by gender, age and history of fracture at single-lag days.

| Lag day | Sex | |  | Age(years) | |  | History of fracture | |
| --- | --- | --- | --- | --- | --- | --- | --- | --- |
|  | Male | Female |  | <75 | ≥75 |  | Yes | No |
| lag0 | 1.38 (0.87,2.20) | 0.93 (0.74,1.17) | | 0.90 (0.67,1.21) | 1.11 (0.84,1.45) | | 1.24 (0.82,1.89) | 0.95 (0.75,1.19) |
| lag1 | 1.24 (0.93,1.66) | 0.96 (0.84,1.11) | | 0.93 (0.78,1.12) | 1.08 (0.92,1.28) | | 1.12 (0.87,1.46) | 0.98 (0.85,1.13) |
| lag2 | 1.13 (0.95,1.33) | 0.99 (0.91,1.07) | | 0.97 (0.87,1.07) | 1.06 (0.96,1.17) | | 1.02 (0.88,1.20) | 1.01 (0.93,1.10) |
| lag3 | 1.03 (0.87,1.23) | 1.02 (0.93,1.11) | | 1.00 (0.89,1.11) | 1.04 (0.93,1.16) | | 0.95 (0.80,1.12) | 1.04 (0.95,1.14) |
| lag4 | 0.96 (0.76,1.23) | 1.04 (0.92,1.17) | | 1.02 (0.88,1.19) | 1.02 (0.88,1.18) | | 0.90 (0.72,1.12) | 1.06 (0.94,1.20) |
| lag5 | 0.92 (0.70,1.21) | 1.05 (0.92,1.20) | | 1.04 (0.88,1.23) | 1.00 (0.85,1.18) | | 0.87 (0.68,1.12) | 1.07 (0.93,1.23) |
| lag6 | 0.90 (0.70,1.15) | 1.05 (0.93,1.19) | | 1.05 (0.91,1.23) | 0.99 (0.85,1.15) | | 0.89 (0.71,1.11) | 1.06 (0.94,1.20) |
| lag7 | 0.90 (0.76,1.08) | 1.05 (0.96,1.15) | | 1.06 (0.95,1.18) | 0.98 (0.88,1.09) | | 0.92 (0.78,1.10) | 1.05 (0.96,1.14) |
| lag8 | 0.92 (0.79,1.08) | 1.04 (0.96,1.12) | | 1.06 (0.96,1.17) | 0.97 (0.88,1.07) | | 0.99 (0.85,1.14) | 1.02 (0.94,1.10) |
| lag9 | 0.95 (0.72,1.25) | 1.03 (0.90,1.17) | | 1.06 (0.89,1.25) | 0.96 (0.81,1.13) | | 1.07 (0.85,1.36) | 0.99 (0.86,1.13) |
| lag10 | 0.99 (0.63,1.55) | 1.01 (0.81,1.25) | | 1.05 (0.80,1.39) | 0.95 (0.73,1.24) | | 1.17 (0.80,1.73) | 0.95 (0.76,1.20) |

**Table S5.** The relative risks with 95%CI in hospitalizations for osteoporotic fractures of cold effect (-2.0℃ vs 25.8℃) at lag 2 day by changing different lag or degree of freedom (df) values for parameters of cross-basis function, calendar time, rainfall, and sunshine duration.

| variable | | parameter | value | RR（95%CI） |
| --- | --- | --- | --- | --- |
| Cb.AT | | lag | 10* | 1.13 (1.04,1.24) |
|  | |  | 9 | 1.13 (1.03,1.23) |
|  | |  | 11 | 1.57 (1.01,2.44) |
|  | | df | 5* | 1.13 (1.04,1.24) |
|  |  | | 4 | 1.14 (1.04,1.26) |
|  | |  | 6 | 1.14 (1.04,1.25) |
| Cb.lag | | df | 3* | 1.13 (1.04,1.24) |
|  | |  | 2 | 1.14 (1.05,1.25) |
|  | |  | 4 | 1.09 (0.95,1.25) |
| time | | df | 1* | 1.13 (1.04,1.24) |
|  | |  | 2 | 1.08 (0.93,1.25) |
|  | |  | 3 | 1.08 (1.00,1.16) |
| rainfall | | df | 3* | 1.13 (1.04,1.24) |
|  | |  | 2 | 1.08 (0.94,1.24) |
|  | |  | 4 | 1.09 (0.95,1.25) |
| sunshine duration | | df | 3* | 1.13 (1.04,1.24) |
|  | |  | 2 | 1.08 (0.94,1.25) |
|  | |  | 4 | 1.09 (0.94,1.25) |

Cb.AT: Cross basis of apparent temperature, Cb.lag: Cross basis of lag.

*The parameter value used in this study model.


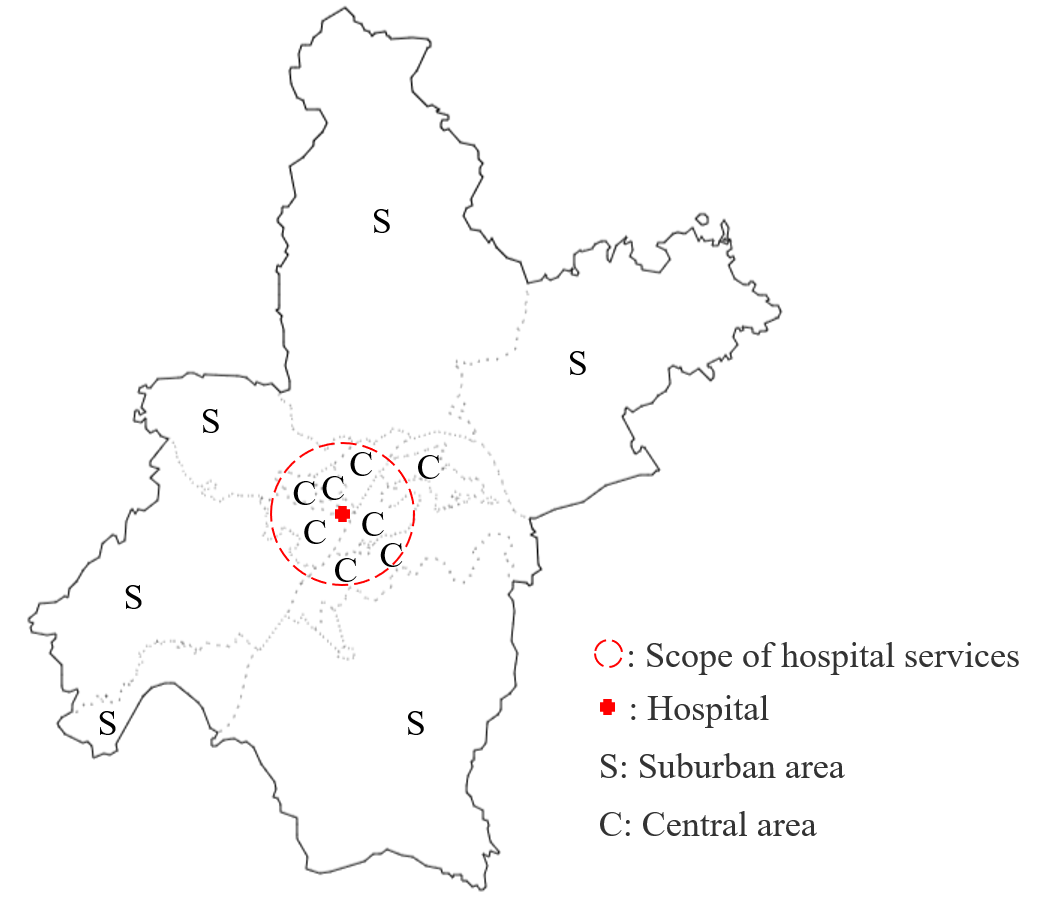


**Figure S1.** The location and main service scope of Wuhan Hospital of Traditional Chinese and Western Medicine


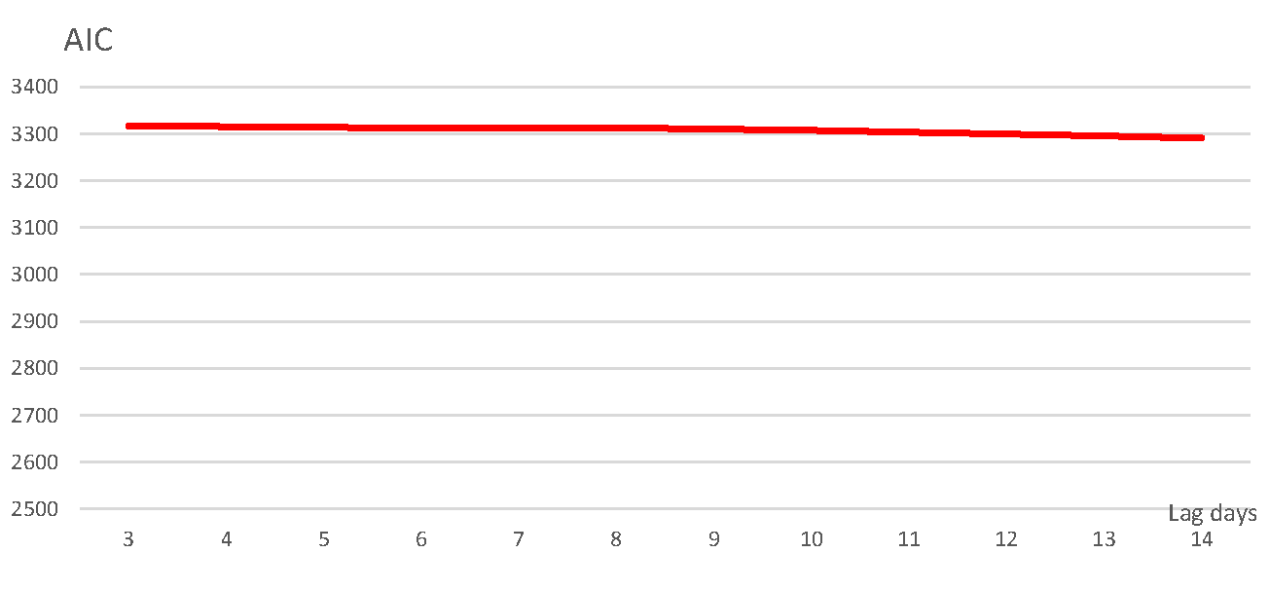


**Figure S2.** The AIC at different lag days


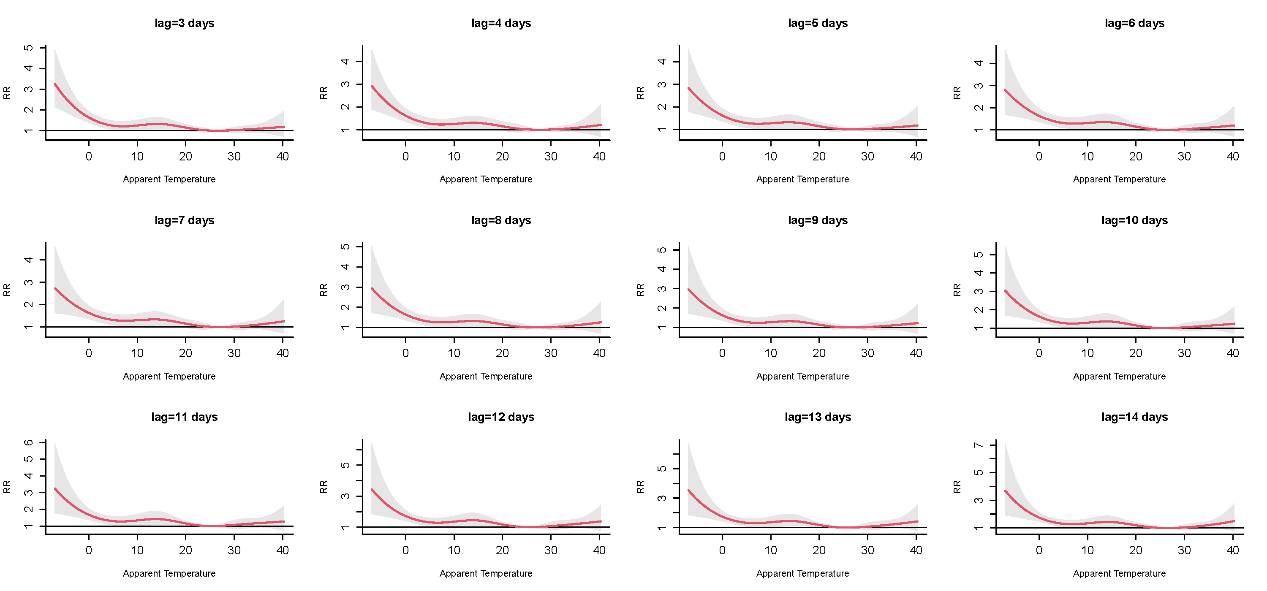


**Figure S3.** Cumulative association and AT distribution of OF at different lag days


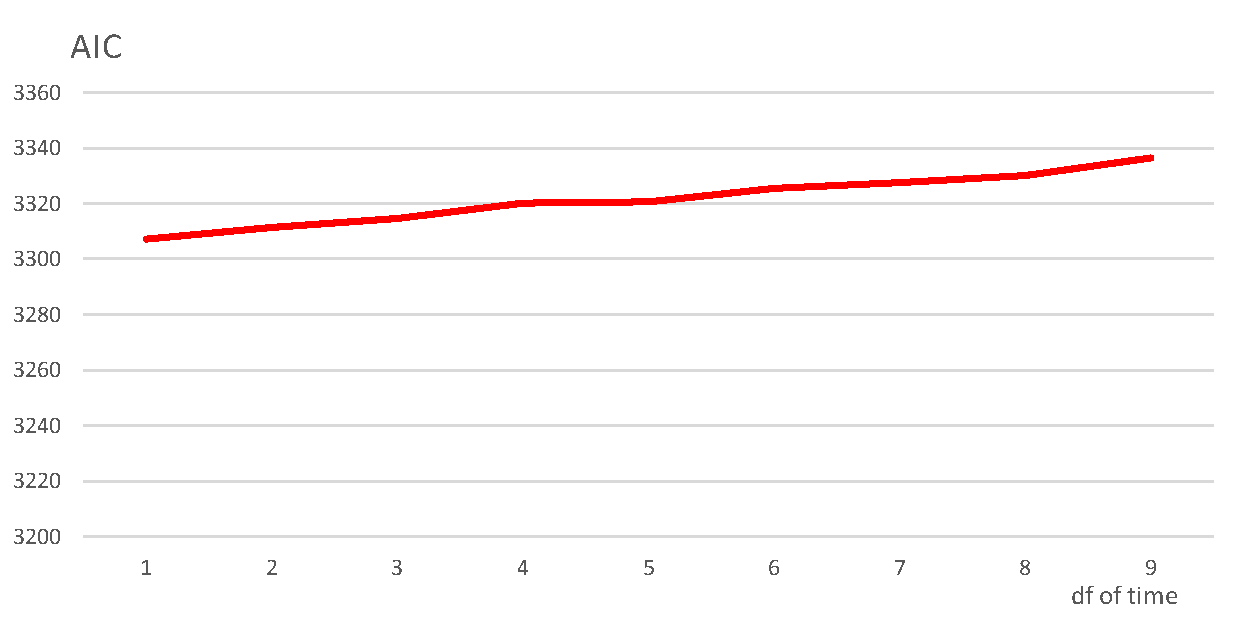


**Figure S4.** The AIC at different df of time


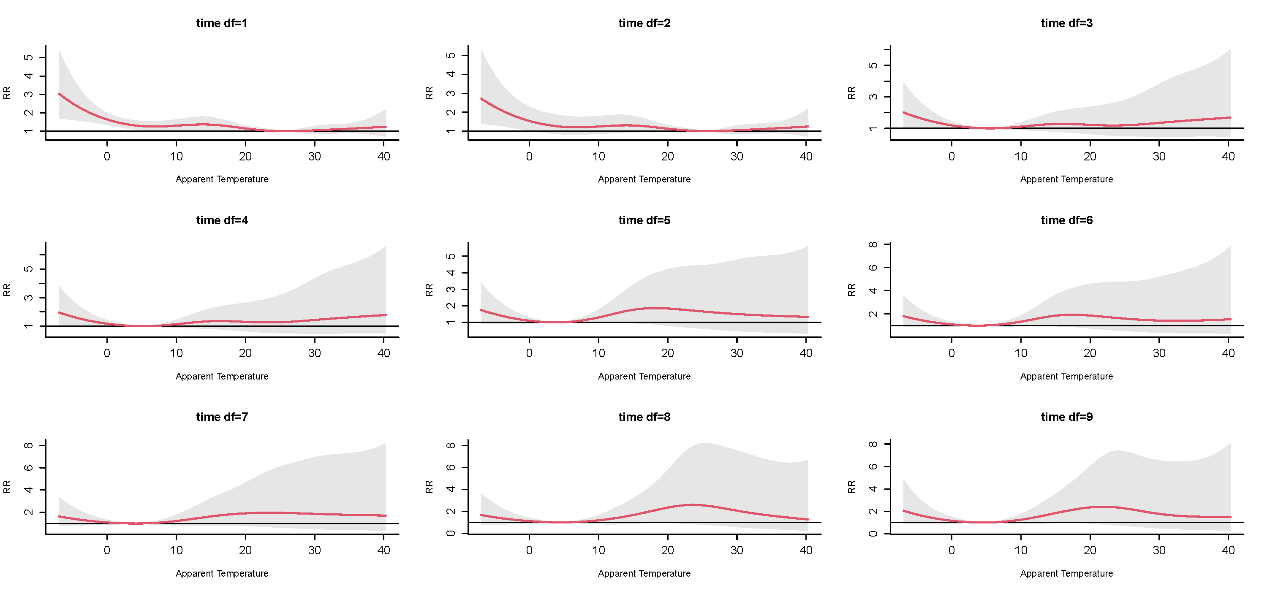


**Figure S5.** Cumulative association and AT distribution of OF at different df for time trend.

**
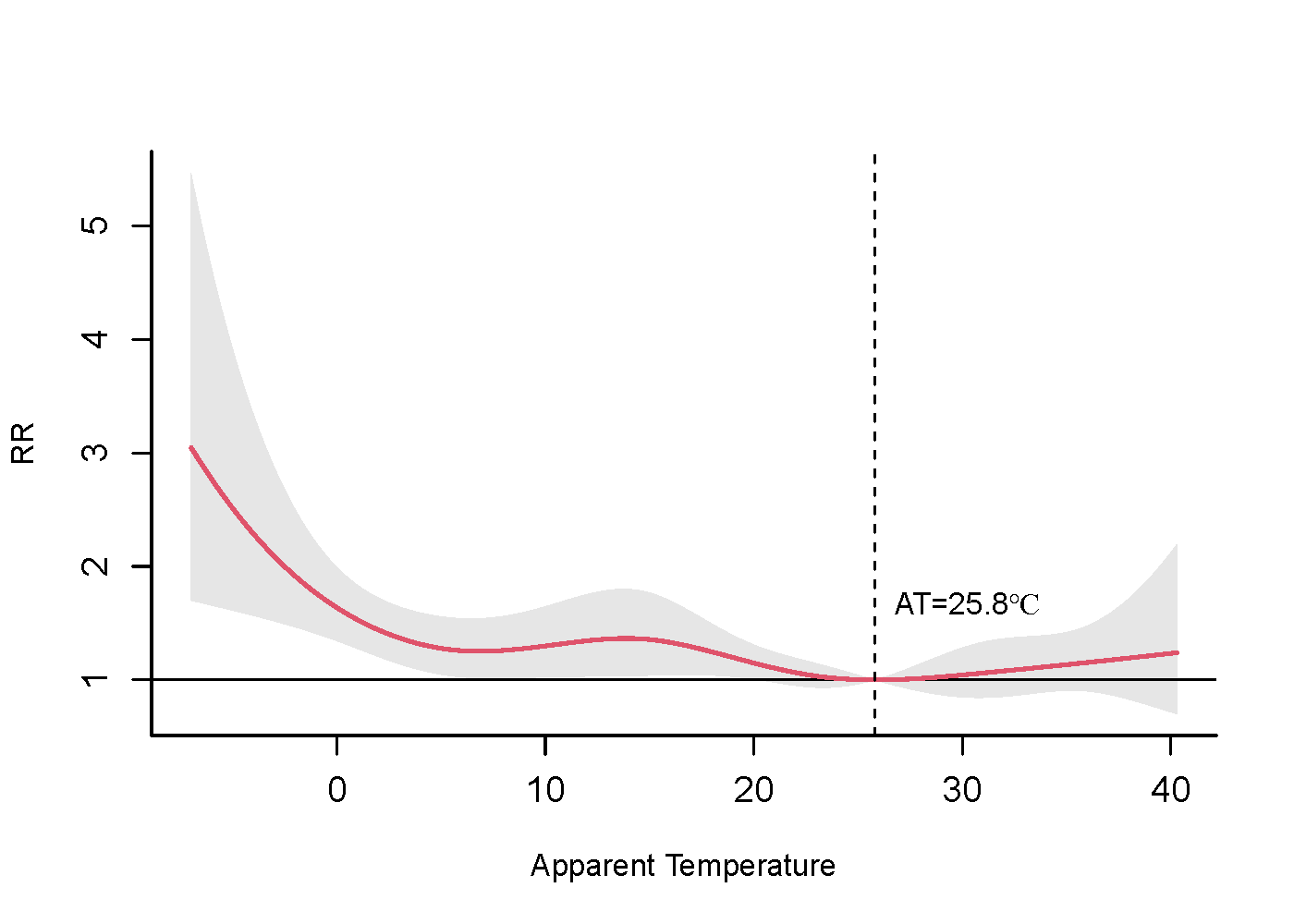
**

**Figure S6.** The exposure-response curve of apparent temperature and the hospitalizations for osteoporotic fractures at lag 0-10 days


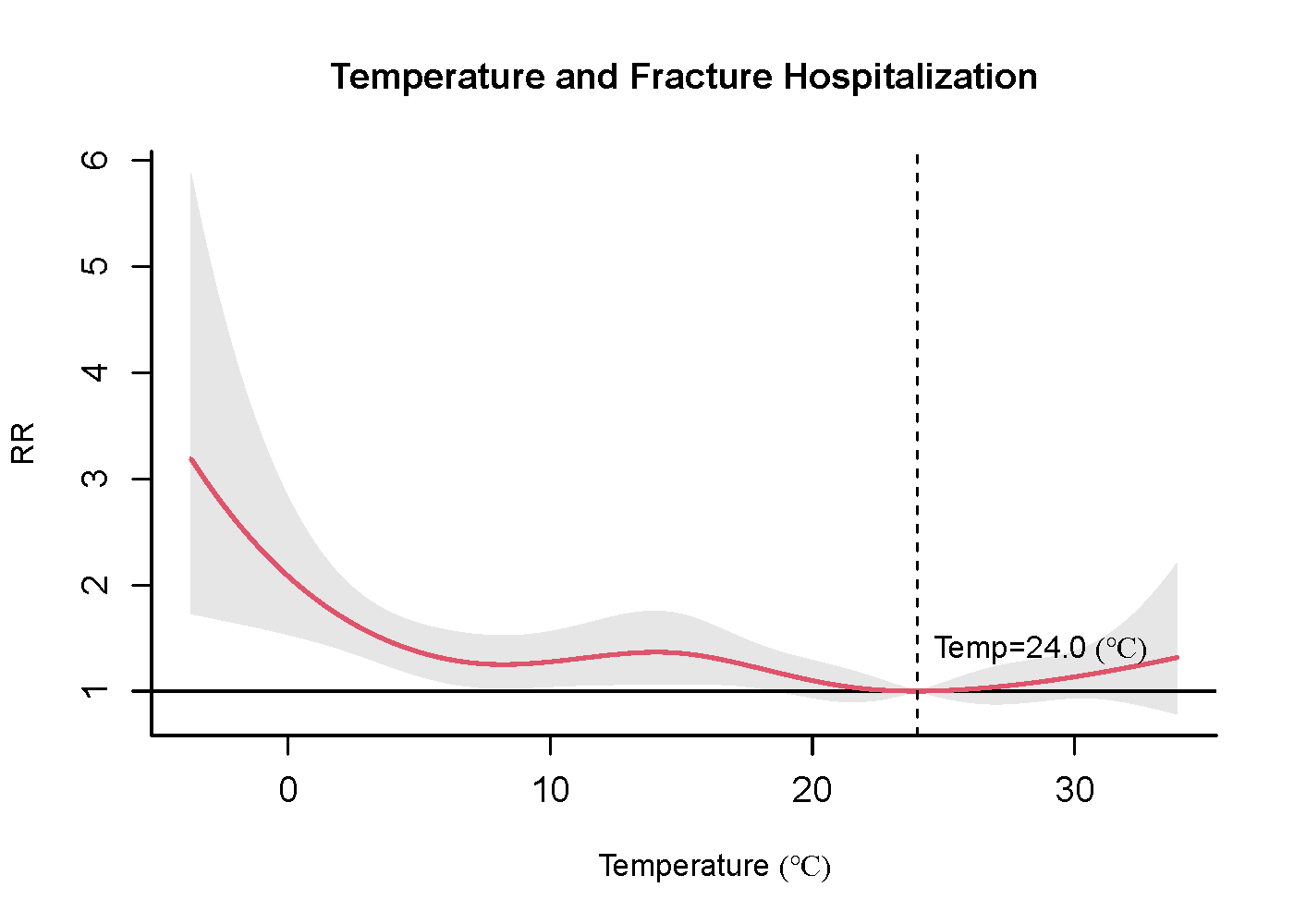


**Figure S7.** The exposure-response curve of absolute temperature and the hospitalizations for osteoporotic fractures at lag 0-10 days


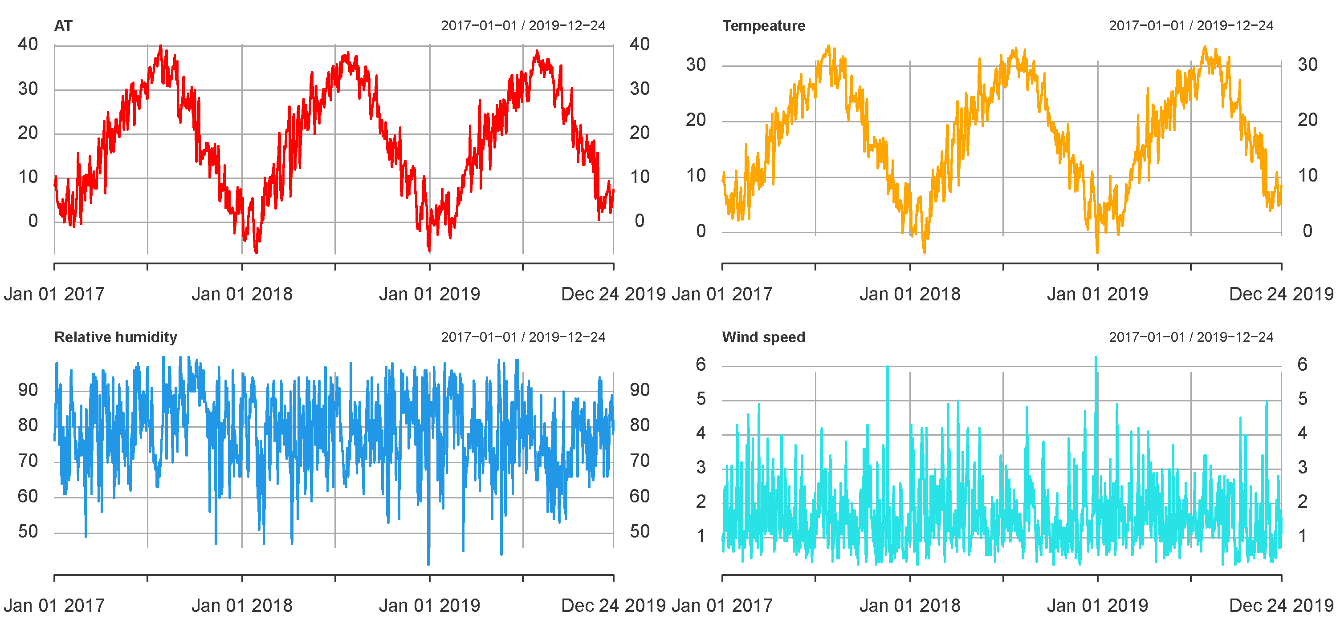


**Figure S8.** The variations of daily meteorological factors from 2017 to 2019 in Wuhan, China.


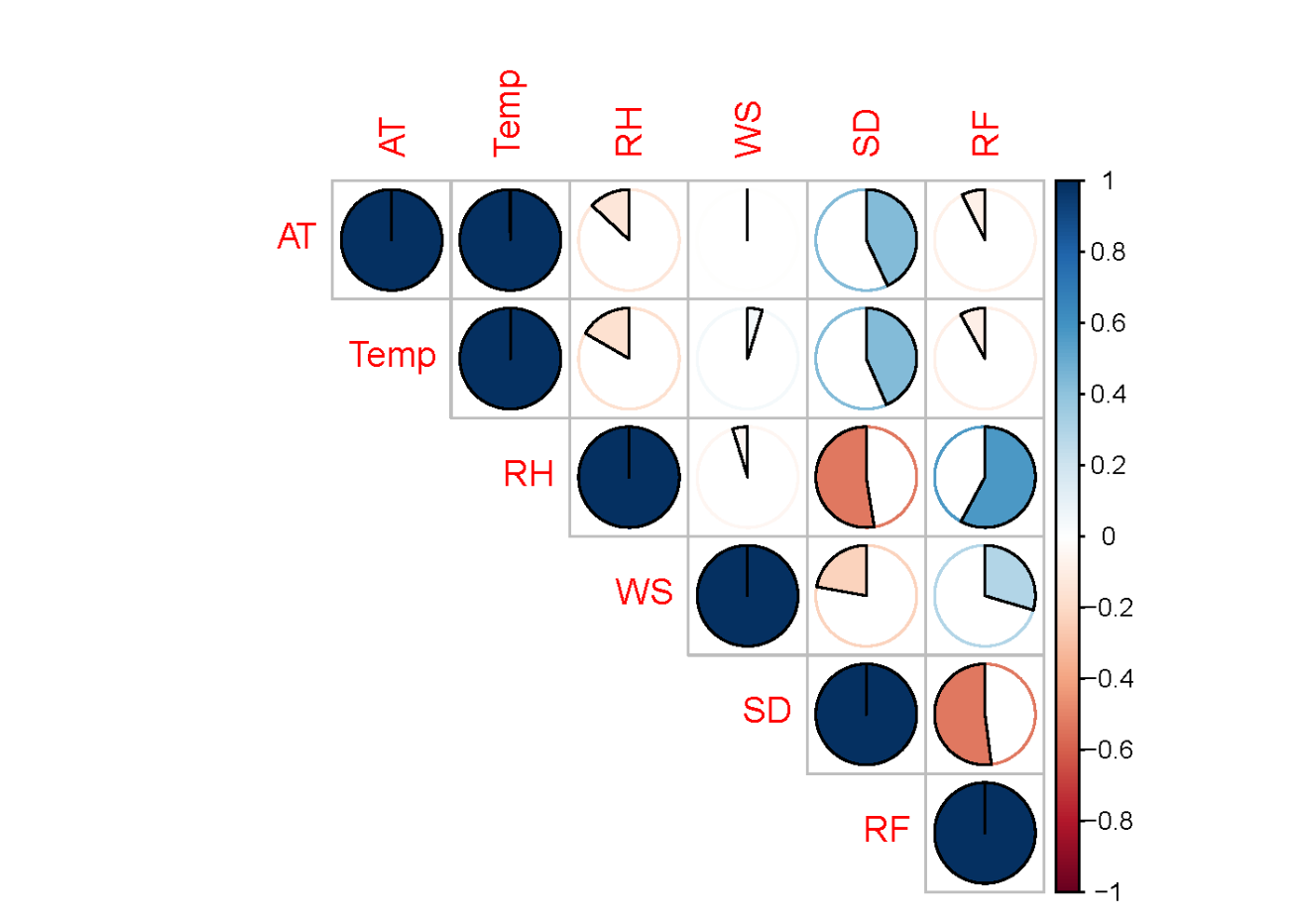


**Figure S9.** The spearman correlation coefficients between meteorological factors.

*AT: apparent temperature, Temp: temperature, RH: relative humidity, WS: wind speed, SD: sunshine duration, RF: rainfall*


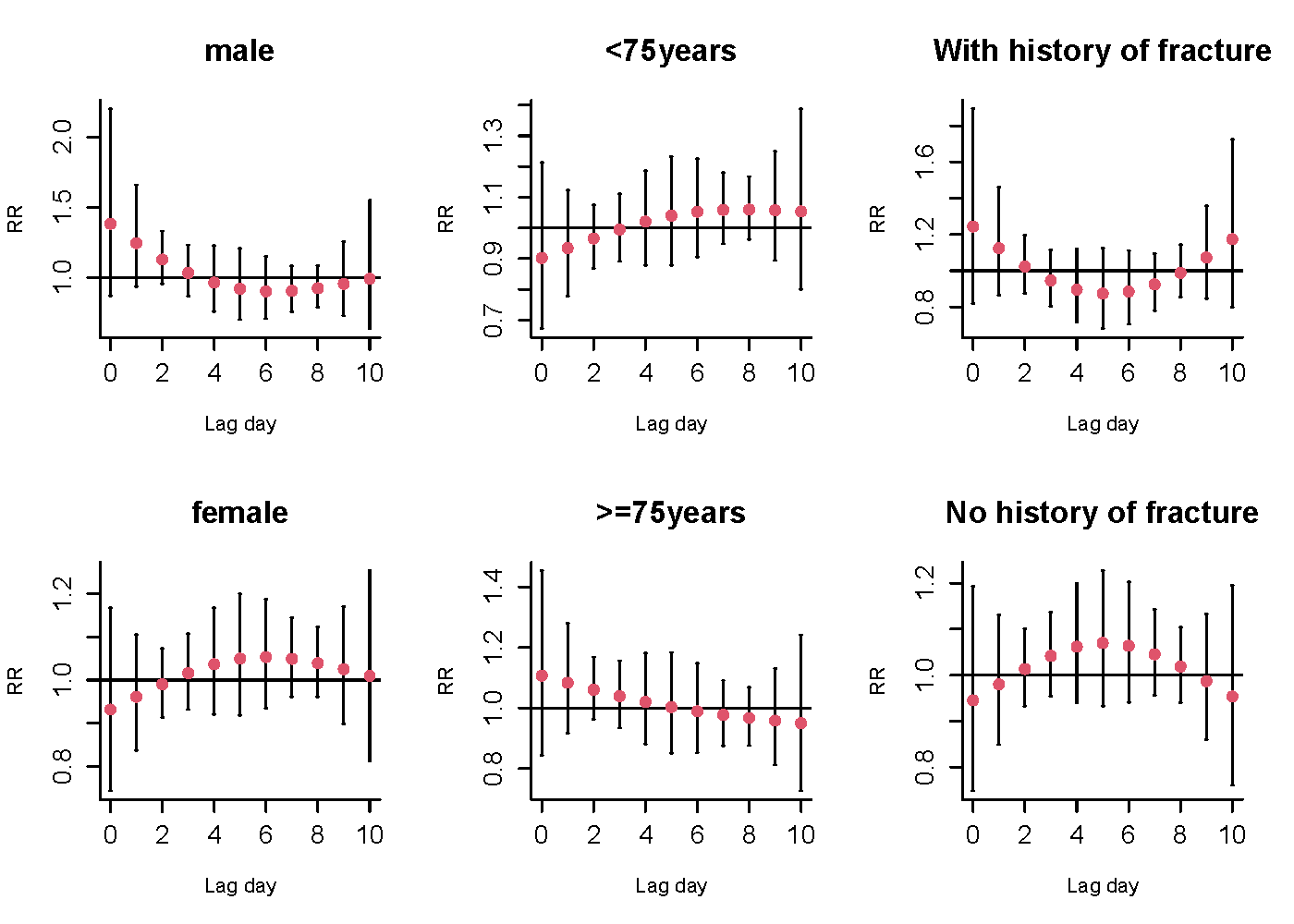


**Figure S10.** The relative risks and 95%CI of warm effect (37℃ vs 25.8℃) on the number of hospitalizations for osteoporotic fractures stratified by gender, age and history of fracture at different lag days
